# MetaAcuPoint: MetaHuman-Generated Synthetic Data for Hand Acupoint Localization

**DOI:** 10.3390/healthcare13233093

**Published:** 2025-11-27

**Authors:** Kasunika Guruge, Prathiksha Padmanabha, H. M. K. K. M. B. Herath, Nuwan Madusanka, Hi-Joon Park, Chang-Su Na, Myunggi Yi, Byeong-il Lee

**Affiliations:** 1 Industry 4.0 Convergence Bionics Engineering, Pukyong National University, Busan 48513, Republic of Korea; kasunikag@pukyong.ac.kr (K.G.); prathikshavp@pukyong.ac.kr (P.P.); kasunkh@pukyong.ac.kr (H.M.K.K.M.B.H.); 2 Digital Healthcare Research Center, Pukyong National University, Busan 48513, Republic of Korea; nuwanv@pknu.ac.kr; 3 College of Korean Medicine, Kyung Hee University, Seoul 02453, Republic of Korea; acufind@khu.ac.kr; 4 College of Korean Medicine, Dongshin University, Jeonnam 58245, Republic of Korea; csna@dsu.ac.kr; 5 Division of Smart Healthcare, College of Information Technology and Convergence, Pukyong National University, Busan 48513, Republic of Korea

**Keywords:** acupoint localization, annotation consistency, deep learning, MetaHuman, synthetic data, virtual reality

## Abstract

Background: Precise localization of acupuncture points (acupoints) is crucial for the clinical success of Traditional Korean Medicine (TKM). Traditional methods that rely on visual inspection and palpation are subjective and prone to inter- and intra-observer differences, making standardization challenging. The progress of data-driven localization techniques is also limited by the scarcity of annotated datasets and inconsistent labeling quality. Objective: This study presents MetaAcuPoint, a synthetic dataset created to overcome these limitations by providing high-fidelity, anatomically consistent hand images for acupoint localization. Methods: MetaAcuPoint was generated using MetaHuman avatars within Unreal Engine, resulting in 900 RGB hand images. Anatomically aligned, bone-attached sockets were implemented for five diagnostically relevant hand acupoints, ensuring millimeter-level precision and spatial consistency across various hand poses. Dataset validity was assessed by training a high-resolution network (HRNet-W48) within the MMPose framework and testing its performance on real-world forearm images. Results: The synthetic-trained model achieved a mean distance error (MDE) of 5.67 ± 3.13 pixels, closely aligning with the real-data baseline at 4.81 ± 2.85 pixels. Adding synthetic samples to real data further enhanced performance (MDE: 4.95 pixels). In contrast, manually annotated synthetic images yielded poorer results (MDE: 12.76 pixels), emphasizing the advantages of automated anatomical annotation. Generalization tests across four external datasets confirmed that the synthetic data-trained model outperformed the real-data-trained model, maintaining higher accuracy (MDE: 5.84–6.45 mm vs. 10.63–15.80 mm). Conclusions: MetaAcuPoint demonstrates the first example of synthetic-to-real generalization for hand acupoint localization. By combining photorealistic rendering with anatomically grounded annotation, the dataset offers a reliable resource to promote standardized, data-driven approaches in acupuncture research and practice.

## 1. Introduction

Acupuncture, a core component of Traditional Korean Medicine (TKM), relies on precisely stimulating anatomically defined sites known as acupoints to modulate physiological functions and restore systemic balance [1,2,3,4,5]. These acupoints are believed to mediate therapeutic effects through neurophysiological pathways and have been used for centuries to treat pain, musculoskeletal disorders, and various systemic conditions [1,2,5,6].

Despite its longstanding use, acupoint localization remains highly subjective. Traditional methods such as visual inspection, palpation, and proportional anatomical measurements (e.g., the cun system) depend heavily on practitioner experience, often resulting in inconsistent outcomes and undermining clinical reproducibility and research reliability [1,3,5,6,7,8,9,10,11]. Such manual techniques usually yield conflicting results, even among trained professionals, which undermines clinical reproducibility and the reliability of research findings [1,6].

Artificial intelligence (AI) and computer vision have been increasingly adopted in clinical workflows to address these limitations. Deep learning (DL), particularly through keypoint detection and pose estimation frameworks, has shown exceptional precision in anatomical landmark localization tasks [12]. Among the available models, the High-Resolution Network (HRNet) stands out for its ability to preserve spatial fidelity, making it particularly suitable for clinically sensitive applications, such as acupoint detection. While real-time models such as RTMPose [13] are promising for dynamic scenarios, this study focuses on HRNet-W48 [5] due to its superior accuracy in prior acupoint benchmarks.

However, the performance of DL models is constrained by the quality, scale, and consistency of training data [5]. Manual annotation of real-world hand acupoint datasets is time-consuming, requires domain-specific expertise, and often lacks demographic and morphological diversity. Moreover, it suffers from substantial inter-annotator variability, resulting in label noise that degrades model performance [1,5,12]. These limitations underscore the pressing need for scalable and reliable annotation strategies, particularly those facilitated by simulation-based methods.

Synthetic data generation offers a practical approach. Unlike traditional augmentation, synthetic datasets are created from scratch using photorealistic simulation engines. Paired with anatomically accurate MetaHuman avatars, tools like Unreal Engine enable researchers to model various hand shapes, poses, and lighting conditions while embedding precise 3D acupoint labels directly into the virtual anatomy [14]. This method allows for consistent, pixel-perfect annotations at scale, providing control over anatomical and demographic variations.

Several studies have validated the utility of synthetic data in pose estimation and medical imaging. For example, Hasan et al. [15] introduced the Hi5 dataset, comprising over 580,000 synthetic hand images, and demonstrated strong generalization to real-world benchmarks. Similarly, Lee et al. [16] developed LightHand99K for wrist-worn camera perspectives, significantly improving model robustness under occlusion and varying viewpoints. These findings underscore the potential of synthetic data to mitigate common limitations in real-world medical datasets. Yet, applying synthetic data to fine-grained, clinically validated tasks, such as acupoint localization, has received limited attention. Previous studies have primarily focused on general joint estimation or used handcrafted features on small-scale datasets [7], without addressing the millimeter-level precision and anatomical specificity required in acupuncture.

**Figure 1:**
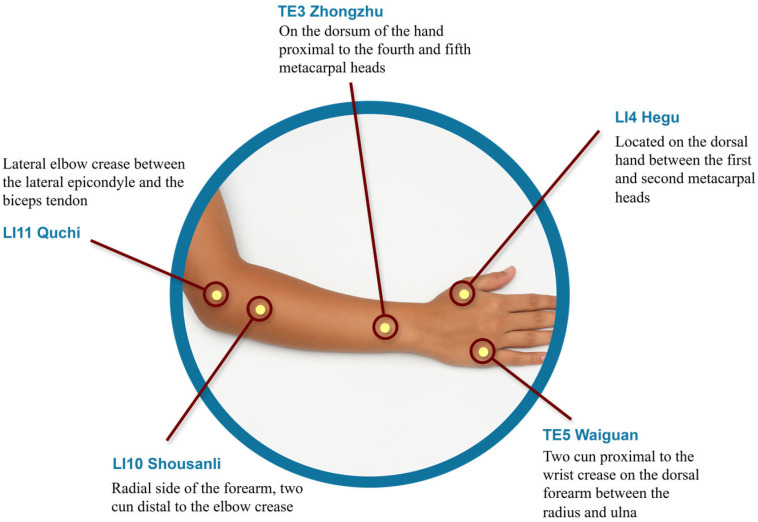
Anatomical locations and descriptions of the five target acupoints used in this study, which are commonly selected in Traditional Korean Medicine for treating upper-limb motor dysfunction in Parkinson’s disease therapy.

We introduce MetaAcuPoint, a high-fidelity synthetic dataset for hand acupoint localization to fill this gap. Using Unreal Engine 5.4 and MetaHuman avatars, we generated 900 RGB images with five diagnostically relevant acupoints: LI4, LI10, LI11, TE3, and TE5. These acupoints were selected for their established clinical relevance in Traditional Korean Medicine, particularly treating upper-limb motor dysfunction in Parkinson’s disease. The initial acupoint locations were determined using standardized anatomical references, specifically the WHO Standard Acupuncture Point Locations in the Western Pacific Region [17] and Focks et al. [18], and verified by a certified TKM practitioner. As illustrated in Figure 1, the five points are anatomically distributed along the forearm and dorsal hand and were embedded as bone-attached sockets within the virtual skeletal mesh to ensure consistent anatomical positioning across diverse avatars and poses. These points were selected to replicate the PK dataset [5] for fair benchmarking. They are also clinically relevant in Traditional Korean Medicine for managing upper-limb motor dysfunction, pain modulation, and rehabilitation after neurological conditions like Parkinson’s disease and stroke. This clinical rationale aligns with previous research on therapeutic acupoint selection for restoring upper-limb function [17,18]. This provides anatomical consistency and reusable annotations across diverse poses and avatars, ensuring a unified experience. This annotation approach departs from previous surface-based or joint-labeling techniques, such as those used in the Hi5 dataset [15], by anchoring keypoints within the bone-local coordinate system of each MetaHuman avatar. As a result, the acupoint locations remain anatomically invariant under pose changes, addressing a critical limitation in synthetic datasets where landmarks may shift with deformation. This consistency is vital in clinical AI tasks that demand high spatial accuracy. We then evaluated HRNet-W48 trained on real-only, synthetic-only, and hybrid datasets to assess model accuracy, annotation consistency, and generalizability to unseen imaging conditions.

This study is a foundational exploration into the viability of synthetic data for acupoint localization. Our primary objective is to evaluate whether high-fidelity, automatically annotated synthetic images can substitute or augment real-world datasets when such data is scarce. To enable a fair, one-to-one comparison, we intentionally designed the synthetic dataset (MetaAcuPoint) to mimic the structure and constraints of an existing, clinically annotated dataset (PK dataset), including a comparable image count (900), five acupoints, a fixed camera angle, and consistent lighting. These constraints are not limitations of the synthetic approach itself, but rather deliberate design choices made to ensure comparability and isolate the impact of data realism and annotation consistency.

In summary, this work offers three primary contributions: (1) A photorealistic, simulation-based dataset with anatomically embedded acupoint labels using MetaHuman and Unreal Engine, (2) A comparative evaluation of HRNet-W48 trained on real, synthetic, and combined datasets for clinical acupoint localization, and (3) Empirical validation that high-quality synthetic data can enhance annotation consistency, generalization, and training stability for scalable clinical AI pipelines.

The rest of this paper is organized as follows: Section 2 reviews related work on deep learning, clinical annotation, and synthetic datasets. Section 3 describes the proposed synthetic data generation pipeline and model training process. Section 4 shares experimental results and analysis. Section 5 wraps up with limitations and suggestions for future research.

## 2. Literature Review

Accurate acupoint identification is fundamental to Traditional Korean Medicine (TKM); however, manual localization remains unreliable due to its subjectivity and dependence on the practitioner’s experience and expertise. Recent advances in deep learning (DL), particularly convolutional neural networks (CNNs), have driven efforts to automate acupoint detection with greater precision and consistency. High-resolution architectures such as HRNet have proven particularly effective, preserving spatial fidelity throughout the network and enabling sub-pixel localization accuracy. This section reviews prior work across four key domains: deep learning-based acupoint localization, limitations of real-world datasets, the use of synthetic data in clinical AI, and simulation-based synthetic datasets for anatomical landmark detection.

The automation of acupoint localization has emerged as a critical frontier in TKM research, aiming to reduce practitioner-dependent variability through DL-based techniques. Seo et al. [5] introduced the PK dataset as a benchmark for hand acupoint localization and demonstrated that HRNet-W48 outperformed ResNet architectures, achieving sub-5-pixel error, surpassing even expert annotators in accuracy. Beyond the hand, Yang et al. [19] proposed a self-attention-enhanced CNN that localized 84 acupoints on the human back with millimeter-level precision, trained on a physician-annotated dataset aligned with national standards. Building on this, Yang et al. [20] developed RT-DEMT, a real-time hybrid model that combined Mamba and Transformer components, reducing inference latency by 14% while maintaining high accuracy.

Additional progress has been made through multistage or cascade approaches. For instance, Sun et al. [10] applied a YOLOv5+HRNet cascade architecture for acupoint detection across broad anatomical regions. Malekroodi et al. [9] further improved real-time performance by integrating anatomical landmarks with CNN-based pose estimation, achieving <5 mm localization error across face and hand regions. Collectively, these studies validate DL’s capacity to achieve high-resolution, scalable, and clinically meaningful acupoint detection.

Despite these advances, real-world datasets face significant constraints in terms of scalability, diversity, and annotation reliability. Annotating acupoints requires domain-specific anatomical expertise, and even expert-generated datasets such as PK [5] are susceptible to inter-annotator variability, leading to millimeter-level discrepancies that can exceed model error margins [21]. This issue is compounded by demographic homogeneity and narrowly defined acquisition protocols. For example, models trained on such constrained datasets often struggle to generalize across external conditions, as shown in studies involving chest X-rays and other clinical imaging domains [22,23].

To address these limitations, synthetic data generation has gained increasing attention as a privacy-preserving and cost-effective alternative to real data. In particular, simulation-driven approaches using photorealistic engines, such as Unreal or Unity, enable precise anatomical modeling and consistent annotation at scale [15,24]. These approaches contrast with GAN-based methods [8,23,25], which, while effective in segmentation and classification tasks, often lack anatomical specificity. Man and Chahl [26] classify synthetic datasets into digitally altered real images versus fully simulated scenes, noting that the latter are better suited for annotation automation. Furthermore, Rujas et al. [27] and Lee et al. [28] emphasize the role of synthetic data in accelerating biomedical research and overcoming regulatory hurdles, such as IRB approval [29], making it an attractive option for the development of clinical AI. Giuffrè and Shung [30] emphasized its role in augmenting datasets for predictive modeling while proposing frameworks like differential privacy to mitigate re-identification risks.

Recent synthetic datasets such as Hi5 [15], AcuSim [4], and LightHand99K [16] have demonstrated strong results in hand pose estimation. For instance, Hasan et al. [15] introduced a synthetic dataset of 580,000 images with varied skin tones and hand articulations, enabling models to outperform real-data baselines. Sun et al. [4] presented AcuSim for cervicocranial acupoint localization, reporting an accuracy of over 92% within a 5 mm margin based on expert review. Similarly, Lee et al. [16] addressed wrist-worn perspectives, enhancing robustness under occlusion. However, these datasets primarily target joint localization or generalized pose estimation rather than clinically validated acupoint detection, and few embed anatomically grounded labels suitable for therapeutic applications.

Several studies have also explored hybrid training strategies. Schülein et al. [31] and Tremblay et al. [32] demonstrate that combining real and synthetic data, particularly under domain randomization, can narrow the performance gap between synthetic and real data. Seib et al. [33] and Kazeminia et al. [8] further argue that mixed datasets improve generalization and reduce annotation costs. Nevertheless, the “reality gap” remains a persistent challenge [34], especially in clinical settings where models may learn superficial cues (“synthetic simplicity”) rather than semantically meaningful patterns [35].

Moreover, few existing works address annotation consistency as a central issue. Many rely on handcrafted features or manual labeling without structured validation [7,10,20], despite findings that annotation noise significantly impairs performance in medical image analysis [12,21]. For instance, even high-performing models like HRNet-W48 [5] or hybrid transformers like RT-DEMT [20] are trained on small-scale, expert-annotated datasets, which limit their reproducibility and scalability. Notably, while synthetic avatars have been used for marker detection in surgical navigation [24] and for real-time acupoint detection [9], most studies lack standardized benchmarks, structured evaluation protocols, or clinical-level error analyses [36].

In summary, synthetic data presents a promising solution to the limitations of real-world datasets; however, current efforts remain fragmented. No previous study fully combines photorealistic simulation, automated acupoint labeling, clinical evaluation, and domain randomization within a single framework. Table 1 provides a comparative overview of key studies on acupoint localization and related synthetic datasets. It highlights differences in dataset sources, annotation methods, model architectures, evaluation metrics, and clinical validation strategies. This structured comparison shows how MetaAcuPoint uniquely combines anatomical accuracy, synthetic data realism, and clinical-level validation within a single framework.

## 3. Methodology

This study utilized two datasets for model training and evaluation: (1) the PK dataset [5], a clinically acquired collection of real hand images previously used as a benchmark, and (2) MetaAcuPoint, a newly developed simulation-based synthetic dataset generated from high-fidelity virtual human models. Both datasets were curated to localize five clinically relevant acupoints (LI4, LI10, LI11, TE3, and TE5).

### 3.1. Dataset Preparation

To evaluate the utility of synthetic data as a substitute or supplement to real clinical data, the MetaAcuPoint dataset was designed to closely mirror the conditions of the PK dataset, which served as the benchmarking standard. This included limiting the number of acupoints to five, matching the total number of images (900), and replicating the camera viewpoint, resolution, background, and lighting. These controls ensured that any observed performance differences reflected the data type (synthetic vs. real) rather than confounding variables.

#### 3.1.1. Material Used

The real dataset used in this study was the PK dataset, developed by Seo et al. [5] and provided by the authors for academic research and comparative evaluation. The dataset comprises 940 RGB images (1488 × 837 pixels) of hands captured under controlled clinical conditions with standardized positioning. Participants placed their forearms and hands flat on a white background. At the same time, a fixed overhead RGB camera acquired top-down images under uniform indoor lighting, producing 10 images per subject (five per hand) with minimal variation in pose.

Manual annotations were performed by trained TKM technicians using the COCO Annotator tool, marking pixel coordinates of five acupoints per hand according to medical anatomical references. As with most manually annotated datasets in clinical AI, the PK dataset is subject to inter- and intra-observer variability. Annotation quality was maintained through technician training and cross-checks, though no automated calibration was applied. In total, 760 images were used for training and 180 for evaluation.

#### 3.1.2. Synthetic Dataset

**Figure 2:**
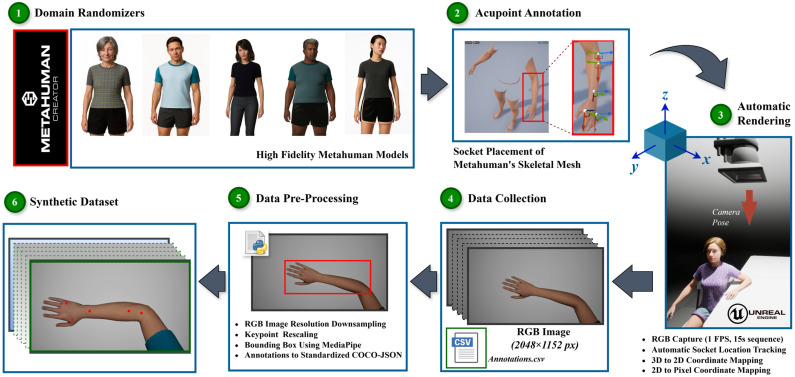
Overview of the synthetic data generation and annotation pipeline. The process includes MetaHuman-based domain randomization, acupoint socket embedding, automatic rendering, 3D-to-2D projection, and COCO-style annotation packaging.

An automated Unreal Engine 5.4 pipeline using MetaHuman was developed to generate annotated synthetic hand images addressing the limitations of clinical datasets. The system enables controlled hand positioning, demographic variation, and reproducible 2D annotations by modeling realistic human hand geometry. The overall data generation and annotation workflow, including domain randomization, acupoint embedding, rendering, and 3D-to-2D mapping, is summarized in Figure 2.

##### Domain Randomization

In this study, domain randomization refers to controlled demographic and structural variations, as well as randomized skeletal morphologies and skin tone distributions, across MetaHuman avatars, while maintaining fixed environmental parameters (lighting and camera) for benchmarking consistency. This partial randomization isolates the effect of morphological diversity from confounding environmental factors, allowing fair comparison with the real PK dataset.

Five skeletal body types (two male, three female) and six avatars per skeleton were used, producing 30 avatars (60 hands; 40% male, 60% female). This balanced scheme prevented overrepresentation of any single morphology. Skin tone diversity was quantified directly from MetaHuman base-color (albedo) textures by sampling the hand region in HSV and RGB color spaces, enabling reproducible appearance variation independent of render lighting. Default MetaHuman presets were retained to match PK dataset conditions and minimize manual bias.

**Figure 3:**
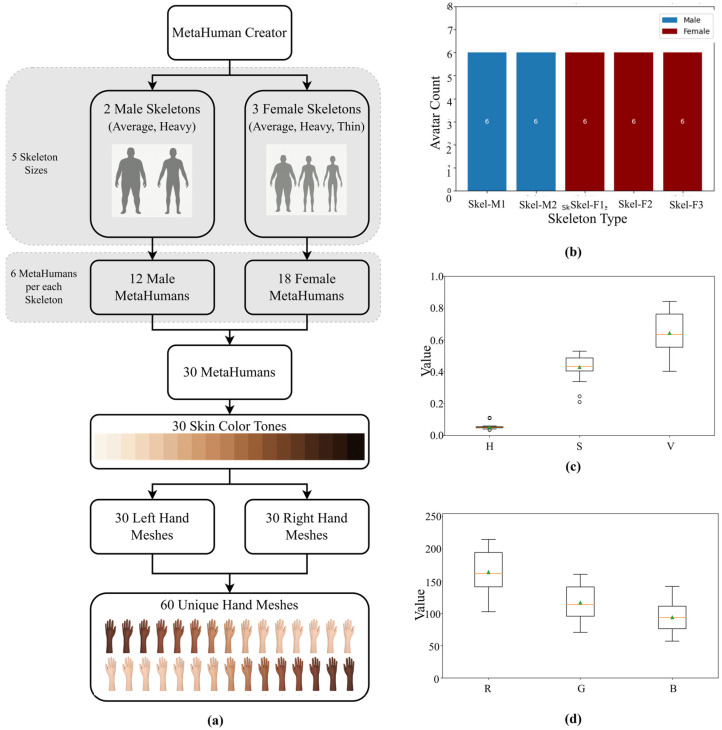
(**a**) Overview of Domain Randomization in the Synthetic Hand Dataset. (**b**–**d**) Statistical characterization of avatar diversity in the MetaAcuPoint dataset. (**b**) Skeleton type distribution: five skeletal body types (Skel-M1, Skel-M2, Skel-F1, Skel-F2, Skel-F3) with equal representation (6 avatars each; 12 males, 18 females). (**c**) Skin tone diversity in HSV color space, showing pooled ranges across 30 avatars: H: [0.0359–0.1120], S: [0.2108–0.5300], V: [0.4035–0.8423]. (**d**) Skin tone diversity in the RGB color space, with channel-specific distributions: R: [102.89–214.78], G: [70.82–161.00], B: [57.07–142.06].

Equal representation across skeleton types and skin-tone ranges enhanced the synthetic dataset’s demographic realism and generalization capacity. Figure 3 summarizes the distribution of skeleton types and skin-tone diversity in HSV/RGB color spaces. Although avatar presets can encode biases (e.g., limited age variation), counts were balanced across body types and tones to mitigate such effects. Age was not explicitly modeled due to limited hand-mesh variability, and this remains a limitation for future dataset iterations. This structured randomization broadened the dataset’s demographic diversity while maintaining experimental control, enabling realistic yet reproducible conditions for downstream model training.

##### Virtual Clinical Environment and Camera Configuration

**Figure 4:**
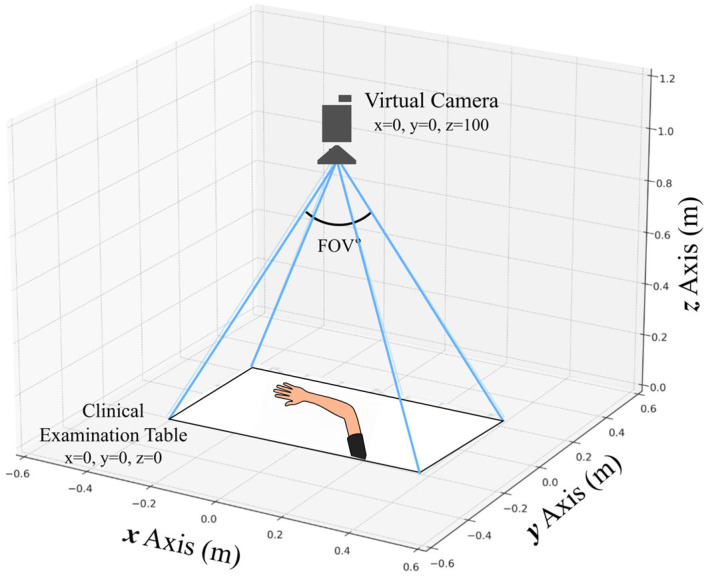
Schematic of the virtual clinical environment and camera configuration used for synthetic image acquisition.

The synthetic capture environment was implemented in Unreal Engine 5.4, utilizing the default MetaHuman Creator assets to maintain consistent geometry, materials, and shading across avatars. The scene replicated the PK dataset’s acquisition geometry, featuring a fixed top-down camera, uniform table placement, and controlled 3 × 3 lighting grid. Table 2 summarizes the scene parameters, camera specifications, lighting configuration, animation ranges, and annotation setup. All parameters remained fixed during dataset generation to preserve spatial alignment and reproducible 3D-to-2D projection. Figure 4 illustrates the virtual camera arrangement and field of view. This deterministic configuration allows exact regeneration of the same 900 images and annotations using the project’s fixed random seed.

##### Acupoint Socket Placement and Replication

To generate precise and frame-consistent 2D annotations without manual intervention, five clinically relevant acupoints (LI4, TE3, TE5, LI10, and LI11) were embedded as invisible sockets within the MetaHuman skeletal mesh. Socket placement followed a two-stage process: (1) expert-guided positioning on representative avatars and (2) automated replication across all models sharing the same skeleton.

For each skeletal type, a certified TKM practitioner manually placed the sockets using standardized anatomical references from the WHO Standard Acupuncture Point Locations [17] within the Unreal Skeleton Editor. Each socket was attached to the appropriate bone (e.g., LI4 on metacarpal_01) and defined in bone-local coordinates to maintain anatomical alignment during hand motion. The resulting transforms were then propagated to all avatars with the same skeletal structure, ensuring positional consistency across morphologies.

Because sockets were embedded in bone-local space rather than on deformable skin meshes, acupoint locations remained anatomically stable throughout motion sequences, enabling accurate 3D-to-2D projection. This approach provides higher spatial precision than joint-based labeling systems (e.g., Hi5 [15]), which focus on skeletal joints rather than clinically defined landmarks. Verification through animation playback confirmed alignment stability across all frames, producing high-fidelity 3D acupoint references for the 900 synthetic images. This socket-based strategy establishes anatomically stable acupoint references, forming the foundation for the 3D-to-2D projection described in the following subsection.

##### Hand Animation and Image Capture

Each of the sixty hand meshes was animated through a unique 15 s Level Sequence within Unreal Engine 5.4. In all sequences, the MetaHuman model was seated with one forearm resting flat on the examination table and the palm fully exposed to an overhead camera. This pose, verified by a TKM expert, ensured unobstructed visibility of the five target acupoints (LI4, TE3, TE5, LI10, and LI11). Controlled articulations were introduced, including wrist and elbow rotations within ±10° and finger flexion/extension within ±10°, to introduce realistic variation without occluding acupoints.

A *CineCameraActor* captured one RGB frame per second (1 fps), resulting in 15 frames of hand. Across 60 hands, the dataset comprised 900 high-resolution images (2048 × 1152 px), each saved as uncompressed PNG files. Instead of manual labeling or mask generation, acupoint annotations were derived from the 3D socket placements via projection, ensuring precision and consistency across all frames.

##### 3D to 2D Coordinate Mapping

Under the fixed top-down orthographic camera setup, all sockets remained constrained to the table plane (z=0) during animation. To generate 2D annotations, the 3D world-space coordinates $p_{world}=(x,y,z)$ of each acupoint were projected into pixel-space coordinates (u,v) using a linear homographic mapping. A one-time calibration determined the camera’s view frustum boundaries on the table surface, based on its physical dimensions. The corresponding coordinate limits ($x_{min},x_{max},y_{min},y_{max}$) defined the table’s extent, while the rendered frame resolution was $R_{w}=2048$ and $R_{h}=1152$ pixels. The pixel-space coordinates were then computed using Equation (1):

(1) $$u\;,\;v=\left(\frac{x-x_{min}}{x_{max}-x_{min}}\right)\cdot R_{w}\;\;\;,\;\;\left(\frac{y-y_{min}}{y_{max}-y_{min}}\right)\cdot R_{h}$$

This formulation accounts for the inversion between Unreal Engine’s world-space y-axis (forward-increasing) and the image-space y-axis (downward-increasing). To match the PK dataset resolution, rendered frames were downsampled by bicubic interpolation to $R_{w}^{\prime }=1488$ and $R_{h}^{\prime }=837$ pixels, and the 2D coordinates rescaled accordingly using Equation (2):

(2) $$\left[u^{\prime },\;v^{\prime }\right]=\left[u\cdot \left(\frac{{R\prime }_{w}}{R_{w}}\right),\;v\cdot \left(\frac{{R\prime }_{h}}{R_{h}}\right)\right]$$

The resulting $\left(u^{\prime },v^{\prime }\right)$ coordinates provided high-precision pixel annotations, exported to CSV files for all 900 synthetic frames.

##### Annotation Packaging

The CSV-based acupoint annotations were converted into a COCO-style JSON format to ensure compatibility with standard keypoint detection frameworks. A single category, *‘hand_acupoint’*, was defined with five ordered keypoints. For each of the 900 synthetic images (1488 × 837 px), metadata fields recorded the image ID, filename, width, and height. The total sample count was closely matched to that of the PK dataset for experimental comparability.

Each annotation entry contained a bounding box (bbox), keypoints, area, and segmentation fields following COCO conventions. The MediaPipe’s hand landmark model was applied to each image to define the hand region automatically, and the resulting bounding box was expanded by 5 px in all directions to ensure full wrist–fingertip coverage. The bbox stored $\left[x_{min},y_{min},\;width,\;height\right]$, with area computed as width × height. Segmentation masks were stored as four-sided polygons. All annotations were compiled into JSON format for direct integration with COCO-compatible training pipelines.

##### Manual Re-Annotation for Consistency Evaluation

To evaluate inter-method consistency, the same 900 set of synthetic images was re-annotated manually by the original team of PK dataset annotators using the COCO Annotator tool. Annotators were blinded to the automatically generated labels. This dual labeling allowed a quantitative comparison between human annotations and the automated pipeline.

### 3.2. Model and Training Framework

**Figure 5:**
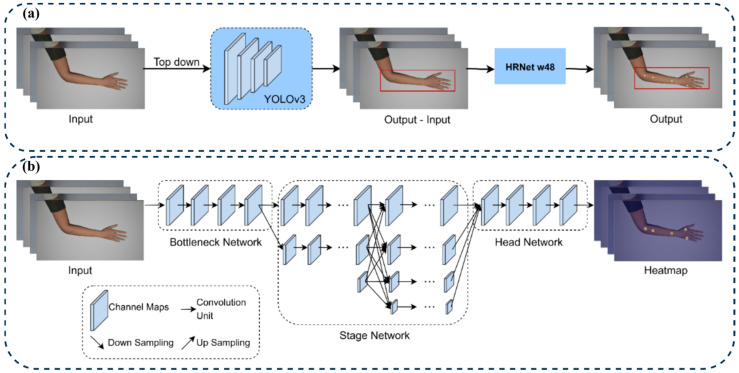
(**a**) The top-down approach inference step and (**b**) the HRNet Model Architecture.

To maintain consistency with the benchmark protocol of Seo et al. [5], the same top-down pose-estimation pipeline was adopted using the MMPose framework with an HRNet-W48 backbone. Hand-bounding boxes were pre-computed using YOLOv3 and supplied to MMPose during both training and inference, replicating the original region-cropping strategy (Figure 5a). Although newer YOLO versions exist, YOLOv3 was retained to ensure direct architectural comparability and because preliminary trials confirmed greater estimation stability for small, high-contrast hand regions.

The framework operates in two sequential stages: YOLOv3 first detects the hand region and generates bounding boxes from the full image; these are then passed to HRNet-W48, which performs keypoint regression (Figure 5b). The top-down design offers higher precision for small structures such as hand acupoints than bottom-up models that process entire images. HRNet-W48 was selected based on prior evaluations that showed a lower mean distance error compared to ResNet and DEKR variants [5]. This choice controls for architectural variability, ensuring that performance differences reflect the characteristics of the model design.

Model training utilized the Adam optimizer (learning rate = 1 × 10^−4^) and the Mean Squared Error (MSE) loss function to minimize the difference between the predicted and ground-truth acupoint coordinates. Each model was trained for 300 epochs with a batch size of 64 and input resolution 256 × 256 px using an NVIDIA RTX 4090 GPU (32 GB RAM). Data augmentation, including random rotation, flipping, and jitter, was applied through MMPose’s integrated pipeline. Early stopping was omitted to maintain consistent epoch counts across datasets.

A quantitative evaluation was performed using the mean Euclidean distance error between the predicted and ground-truth pixel coordinates of the five annotated acupoints. For each acupoint *k* in image *i*, accuracy $D_{k}$ was computed with Equation (3).

(3) $$D_{k}=\frac{1}{N}\sum_{i=1}^{N}\left\|{\hat{P}}_{i}^{k}\right.-{\left.P_{i}^{k}\right\|}_{2}$$

To capture the variability in prediction accuracy, the standard deviation of the Euclidean distance error for each acupoint *k*, denoted as *σ_k_*, was also computed by Equation (4).

(4) $$\sigma_{k}=\sqrt{\frac{1}{N}\sum_{i=1}^{N}{\left(\left\|{\hat{P}}_{i}^{k}-P_{i}^{k}\right\|-D_{k}\right)}^{2}}$$

The overall mean distance error *D* across all *K* acupoints is calculated using Equation (5).

(5) $$D=\frac{1}{K}\;\sum_{k=1}^{K}D_{k}$$

Reporting both mean and standard deviation of localization errors provides a comprehensive measure of model precision and consistency, directly comparable with Seo et al.’s [5] benchmark results, where HRNet-W48 achieved a mean error of 4.81 px. These configurations established a controlled baseline for subsequent experiments comparing real and synthetic training data.

### 3.3. Experimental Setup

Four structured experiments were designed to systematically evaluate the utility of synthetic data for the localization of acupoints. These experiments investigated whether synthetic data could substitute for real data, serve as an effective augmentation, enhance annotation consistency, and support generalization across different imaging viewpoints. All experiments used the same model architecture, training configuration, and a fixed 180-image test set from the real PK dataset to ensure direct comparability.

**Figure 6:**
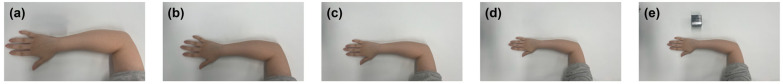
Representative images from the four camera-height test sets used in Experiment 4: (**a**) 30 cm, (**b**) 40 cm, (**c**) 50 cm, and (**d**) 60 cm. The calibration object (**e**) is used to establish pixel-to-millimeter conversion.

- Experiment 1: Evaluated synthetic data as a substitute for real data. The HRNet-W48 model was trained exclusively on 760 synthetic images and then tested on the real PK set to establish a baseline for substitutability.
- Experiment 2: Explored the augmentation effect by combining 760 real + 760 synthetic images (1520 total) in training. This configuration assessed whether synthetic samples enhance model performance by increasing diversity while preserving the distribution of real data.
- Experiment 3: Assessed annotation consistency by comparing models trained on automatically annotated versus manually re-annotated synthetic data. Both models were trained and tested under identical conditions to isolate the effects of labeling on localization accuracy.
- Experiment 4: Examined model generalizability under varied camera viewpoints. Four additional real-world test sets were collected under institutional ethical clearance (IRB No. 1041386-202207-HR-41-02), at camera heights of 30 cm, 40 cm, 50 cm, and 60 cm above the table surface. All other factors (lighting, pose, background) were held constant. A calibrated object in each scene enabled precise pixel-to-millimeter conversion. Two pre-trained models (1:1 training of real/synthetic data) were evaluated without fine-tuning. Figure 6 illustrates representative viewpoints showing field-of-view variation and hand-size reduction.

These experiments were intentionally constrained to a controlled framework to isolate the effects of data source and annotation method on model performance. While Experiments 1–3 focused on the PK benchmark setup, Experiment 4 introduced a new real-world dataset to evaluate cross-view generalization without confounding variables (e.g., occlusion, dynamic gestures). This design provides a clear baseline for out-of-distribution evaluation, with broader generalization scenarios (occlusion, motion) planned for future studies as the dataset expands. This setup enabled controlled cross-view evaluation, as summarized in Table 3.

## 4. Results and Analysis

The proposed MetaAcuPoint (MAP) dataset was evaluated through a series of quantitative and qualitative experiments designed to assess its accuracy, robustness, and clinical relevance. Using the HRNet-W48 architecture, models were trained on real (PK), synthetic (MAP), and combined (PK + MAP) datasets. Performance was compared using mean distance error (MDE) at five acupoints, supported by statistical and visual analyses. Additional experiments examined annotation consistency, acupoint-specific variability, and generalization to unseen camera viewpoints. Together, these analyses evaluate whether high-fidelity synthetic data can (1) achieve accuracy comparable to real-world datasets, (2) complement limited clinical samples through hybrid training, and (3) provide a reproducible and geometry-consistent foundation for acupoint localization.

**Figure 7:**
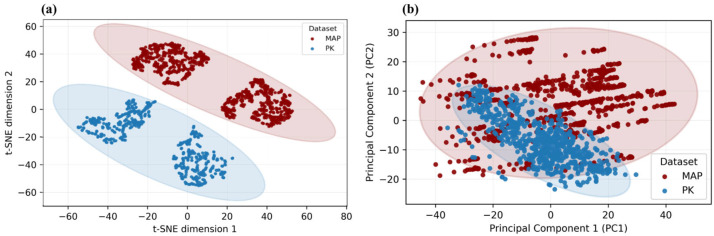
(**a**) t-SNE projection of hand-pose key points showing greater spatial dispersion in MAP than in PK, indicating broader pose variability. (**b**) PCA of skin-tone distributions demonstrating enhanced coverage of chromatic variance in MAP. These analyses collectively validate the dataset’s anatomical and visual diversity.

The diversity of the proposed MetaAcuPoint (MAP) dataset was first examined to verify that it encompasses sufficient anatomical and visual variation for robust model training. As shown in Figure 7, MAP exhibits broader dispersion in both pose and skin tone spaces compared to the real PK dataset, confirming its enhanced coverage of structural and appearance variability. This variability ensures that MAP captures a broader range of realistic limb postures and visual contexts, thereby addressing the data scarcity and homogeneity typically found in clinical image collections. Such diversity underpins the dataset’s capacity for better generalization in subsequent localization experiments.

**Figure 8:**
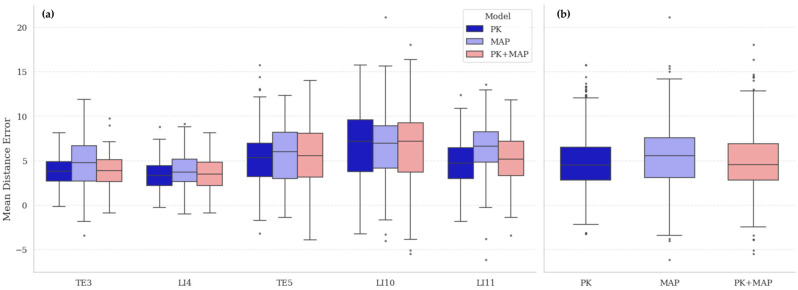
Comparison of localization accuracy across training datasets: (**a**) Point-wise mean distance error (MDE) distributions for five acupoints (TE3, LI4, TE5, LI10, LI11) across models trained on real (PK), synthetic (MAP), and hybrid (PK + MAP) datasets. Consistent performance patterns are observed, with higher variability at LI10 and LI11 due to anatomical complexity. (**b**) Overall MDE distribution across models, illustrating comparable accuracy of MAP and PK + MAP relative to the PK benchmark. The hybrid model maintains the stability of real-data performance while benefiting from synthetic augmentation.

The comparative performance of models trained on real (PK), synthetic (MAP), and hybrid (PK + MAP) datasets is summarized in Table 4 and visualized in Figure 8. All models were evaluated on the real PK test set to establish a consistent reference benchmark. The PK model, trained and tested within the same real-data domain, achieved the lowest overall mean distance error (MDE) of 4.81 ± 2.85 px, reflecting its advantage under in-domain evaluation. The MAP-only model, trained entirely on synthetic data, yielded a slightly higher MDE (5.67 ± 3.13 px) but remained close to the real-data baseline, confirming that MetaAcuPoint captures the geometric and visual properties of real acupoint structures with high fidelity. The hybrid model (PK + MAP) achieved 4.95 ± 2.94 px, indicating that augmenting limited real data with synthetic samples preserved real-domain accuracy while improving prediction stability across acupoints. Statistical analysis confirmed a significant main effect of dataset composition (*F* = 37.15, *p* < 0.001), with pairwise comparisons showing that MAP performed marginally below PK (*p* < 0.01), while PK + MAP was statistically comparable to PK (*p* = 0.31) and significantly better than MAP (*p* < 0.01).

Point-wise comparison revealed consistent acupoint-dependent trends across all configurations. Errors were lowest at TE3 and LI4, moderate at TE5, and highest at LI10 and LI11, patterns that align with known anatomical complexity. TE3 and LI4 lie near bony landmarks providing clear geometric reference cues, whereas LI10 and LI11 are located on the forearm’s extensor surface, where soft-tissue curvature and muscle contraction vary with pose. The repetition of this pattern across datasets indicates that the variations arise from intrinsic anatomical difficulty rather than from dataset artifacts. Overall, these findings confirm that high-fidelity synthetic data can reproduce real-data performance characteristics when evaluated under real-domain conditions, providing a robust foundation for subsequent generalization analysis.

**Figure 9:**
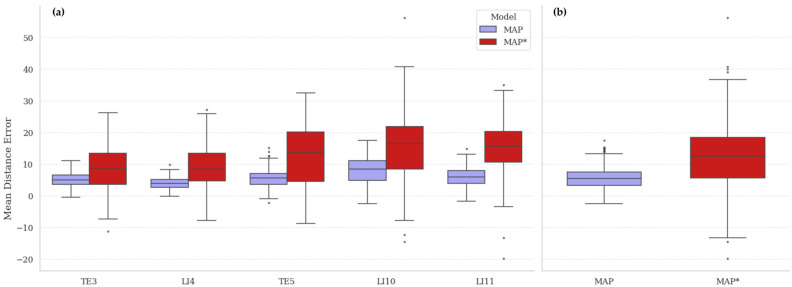
Comparison of annotation consistency between automatically generated (MAP) and manually adjusted (MAP*) synthetic datasets: (**a**) Point-wise mean distance error (MDE) distributions for five acupoints, showing that MAP maintains lower variance across all points. (**b**) Overall MDE distributions highlight the increased dispersion in MAP*, indicating greater human-induced variability. These results demonstrate that automatic procedural labeling ensures more stable and geometrically coherent annotations than manual correction, consistent with inter-annotator variability observed in real clinical datasets.

The consistency of synthetic annotations was evaluated by comparing models trained on the automatically generated MetaAcuPoint (MAP) labels and the manually adjusted synthetic dataset (MAP*). As shown in Figure 9 and the final row of Table 4, the MAP model achieved lower mean distance errors and tighter error distributions across all five acupoints, whereas MAP* exhibited noticeably higher variance and mean errors—particularly at LI10 and LI11. This indicates that human intervention introduced geometric inconsistency rather than improving precision. The superior uniformity of MAP annotations reflects the procedural rig’s ability to generate repeatable and anatomically coherent point coordinates without subjective bias.

To contextualize this finding, the mean inter-annotator deviation in the real PK dataset was approximately 4.8 px, comparable to the deviation observed between MAP and MAP*. This suggests that annotation variability is not a limitation of synthetic imagery but an inherent challenge of manual labeling, consistent with prior observations in clinical datasets [5]. Consequently, these results validate that automated procedural labeling yields higher internal consistency than manual annotation, providing a reproducible and objective foundation for model training.

To evaluate real-world generalization, the models trained on real (PK) and synthetic (MAP) datasets were tested on a separate real dataset captured under four different camera heights. As summarized in Table 5, the MAP-trained model consistently achieved lower mean distance errors (MDEs) across all viewpoints, ranging from 5.16 ± 1.19 mm to 6.45 ± 1.69 mm, whereas the PK-trained model showed substantially higher and more variable errors (10–16 mm). Statistical analysis (Table 6) confirmed a significant height-dependent effect for PK (F = 92.6, *p* < 0.001), but only minor variation for MAP (F = 3.42, *p* = 0.017), indicating that synthetic training produced near-invariant performance across viewing conditions.

**Figure 10:**
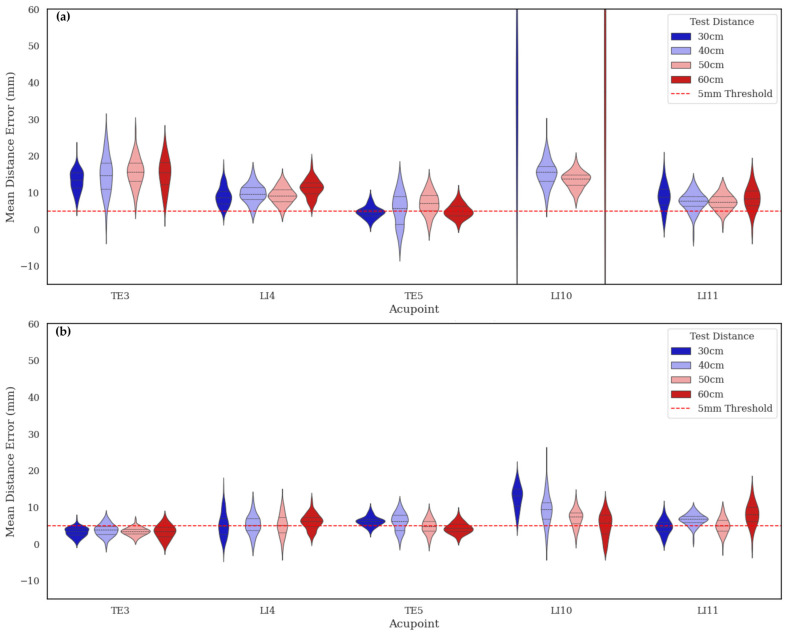
Camera-height generalization performance of PK and MAP models: (**a**) PK model showing strong height-dependent error variation, with deviations exceeding the 5 mm clinical threshold at extreme viewpoints. (**b**) MAP model demonstrating consistent localization accuracy across all camera heights, with most predictions remaining within the 5 mm tolerance (red dashed line). These results confirm that the MAP-trained model achieves viewpoint-invariant generalization while maintaining clinically acceptable precision.

The violin plots in Figure 10 further demonstrate this robustness: MAP maintained consistent error distributions across all camera heights, while PK errors increased significantly at 30 cm and 60 cm, due to steeper view angles and foreshortened limb regions. This demonstrates that the structured variability in the MetaAcuPoint dataset enables models to learn pose- and viewpoint-invariant features, allowing them to effectively adapt to new imaging conditions not encountered during training.

From a clinical perspective, all MAP predictions remained closer to the 5 mm tolerance, the precision threshold generally considered acceptable for automated acupuncture point detection [4]. This accuracy falls well within the 0.5 cun (≈8–10 mm) margin defined by the WHO Standard Acupuncture Point Locations [17], corresponding to practitioner-level manual localization precision. Hence, the synthetic model not only generalizes effectively to real, unseen conditions but also achieves clinically meaningful accuracy, reinforcing its potential as a reliable augmentation source for data-limited clinical applications.

**Figure 11:**
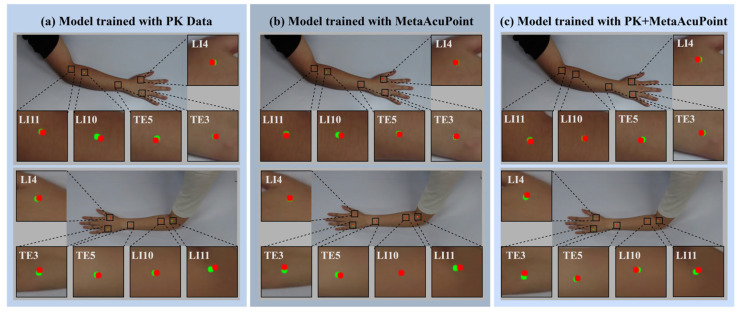
Qualitative comparison of acupoint localization performance: (**a**) PK-trained, (**b**) MAP-trained, and (**c**) PK + MAP-trained models visualized with predicted (red) and ground-truth (green) acupoints. MAP and hybrid models show tighter spatial correspondence, particularly at challenging sites (LI10, LI11), highlighting improved anatomical precision and reduced localization bias.

Qualitative visualizations further substantiate the quantitative results, highlighting the spatial precision and robustness achieved through synthetic and hybrid training. As shown in Figure 11, the model trained on real data (PK) occasionally exhibits lateral or vertical drift at complex anatomical sites such as LI10 and LI11, where muscle curvature and skin folding introduce visual ambiguity. In contrast, the MAP-trained and PK + MAP models demonstrate markedly improved alignment between predicted (green) and ground-truth (red) acupoints, maintaining consistent accuracy across both hand and forearm regions. The hybrid configuration, in particular, shows the tightest alignment, suggesting that augmenting real data with synthetic samples effectively reduces anatomical bias and improves geometric consistency.

**Figure 12:**
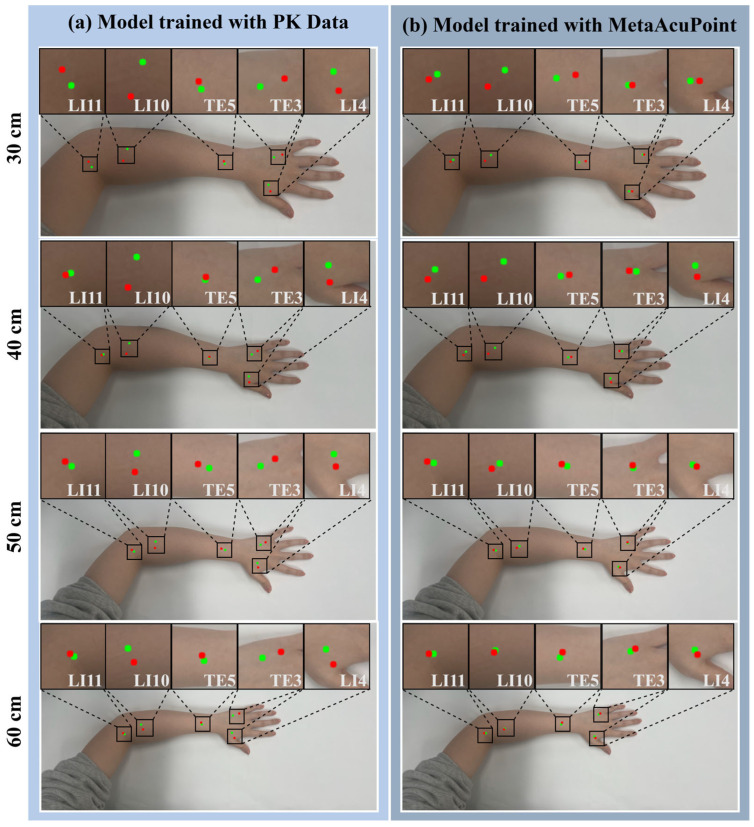
Cross-view generalization under varying camera heights (30–60 cm) visualized with predicted (red) and ground-truth (green) acupoints: (**a**) PK-trained and (**b**) MAP-trained models evaluated on unseen real images. MAP retains consistent acupoint localization across all viewpoints, whereas PK exhibits increased displacement at extreme angles. These results confirm that synthetic pose diversity fosters viewpoint-invariant generalization.

Under viewpoint variation (Figure 12), the PK-only model displays increasing misalignment as the camera height deviates from the training perspective, with noticeable displacements at 30 cm and 60 cm. Conversely, the MAP-trained model retains high localization fidelity across all viewpoints, confirming that the structured pose diversity encoded in the synthetic dataset enables viewpoint-invariant learning. This observation reinforces the statistical findings in Table 5 and Table 6, demonstrating that exposure to controlled synthetic variation enhances spatial robustness without reliance on large real-world samples.

**Figure 13:**
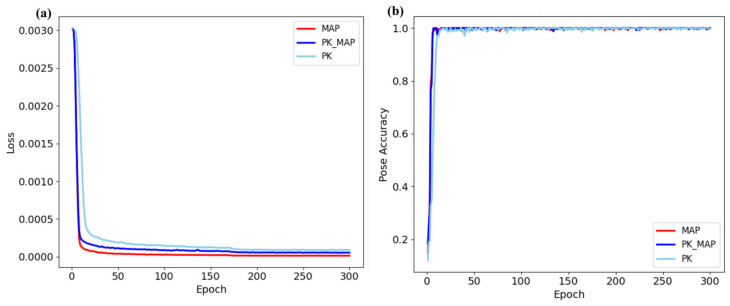
Training dynamics for PK, MAP, and PK + MAP models: (**a**) Loss curves and (**b**) pose accuracy over 300 epochs. The hybrid (PK + MAP) model demonstrates the smoothest and fastest convergence with minimal overfitting, confirming the stabilizing effect of synthetic data augmentation during training.

Training dynamics (see Figure 13) reveal consistent convergence behavior across all models, with notable improvements in stability in synthetic and hybrid configurations. The PK + MAP model achieves the smoothest loss decay and the lowest final loss value (~0.00017), while the MAP-only model converges gradually yet stably. In contrast, the PK-only model exhibits slower convergence and greater loss oscillation, indicating susceptibility to overfitting due to the limited diversity of the data. Collectively, these trends confirm that incorporating structured synthetic data improves both optimization stability and generalization efficiency, supporting the proposed framework’s scalability for clinically relevant acupoint detection.

Collectively, the results demonstrate that the proposed MetaAcuPoint (MAP) framework achieves near–real-world accuracy, enhanced label consistency, and superior generalization across viewpoints and imaging conditions. The integration of high-fidelity synthetic data not only compensates for the scarcity of clinical samples but also improves robustness and reproducibility beyond what is attainable with manual annotation alone. These outcomes confirm that structured synthetic data generation can serve as a reliable and clinically relevant foundation for advancing automated acupoint localization systems.

## 5. Discussion and Conclusions

This study investigated the efficacy and generalizability of synthetic data for acupoint localization by introducing the MetaAcuPoint, a high-fidelity dataset generated using MetaHuman avatars in Unreal Engine 5.4 and validated through controlled experiments against a real clinical benchmark (PK dataset). The central hypothesis proposed that a well-engineered synthetic dataset, designed to address limitations in real-world data such as limited pose variability, restricted skin tone diversity, and inconsistent annotations, can effectively substitute for or augment real data to facilitate robust model training. The experimental results strongly support this hypothesis across all evaluation axes.

The results demonstrate that MetaAcuPoint successfully enhances model generalization while maintaining benchmark-level accuracy. The HRNet-W48 model trained solely on synthetic data achieved an MDE of 5.67 pixels, closely matching the 4.81-pixel benchmark achieved with real data. Despite this marginal difference, the synthetic-trained model exhibited superior adaptability under unseen imaging conditions, particularly across varying camera viewpoints. These outcomes highlight the value of controlled diversity by systematically varying morphology and skin tone while holding environmental factors constant. MAP promotes model robustness to domain shifts. When synthetic and real datasets were combined, model performance improved further (MDE = 4.95 pixels) without loss of accuracy, confirming the value of MAP as a cost-effective augmentation strategy. This empirical evidence suggests that synthetic data can not only substitute for but also complement real data, doubling the dataset size and variability without requiring additional clinical collection or manual labeling. Annotation experiments revealed that automated bone-anchored labeling significantly outperformed manual re-annotation (MDE = 12.76 pixels for manual vs. 5.67 for automated). This finding underscores the challenge of inter- and intra-observer variability in manual labeling and validates the reliability of anatomically embedded, socket-based auto-annotation. Notably, annotation noise varied across landmarks, with higher error near curved or visually ambiguous anatomical regions, an insight that reinforces the importance of anatomically constrained automation for reproducible supervision in medical AI. Qualitative analyses corroborated these quantitative results. Predicted acupoints from synthetic and hybrid-trained models displayed strong anatomical plausibility, with improved spatial alignment and smoother convergence patterns during training. Models trained on synthetic data also exhibited reduced overfitting tendencies, reflecting the stabilizing influence of controlled diversity and precise structured annotation.

MAP represents a scalable, reproducible, and ethically unencumbered framework for creating biomedical datasets. By combining photorealistic modeling, anatomical embedding, and procedural randomization, the pipeline effectively addresses key challenges in clinical AI dataset development, including consistency of annotations, diversity, and data accessibility. Annotation consistency is ensured through bone-local socket labeling, which provides pixel-level reproducibility and eliminates inter-observer variability. Diversity and generalization are enhanced through controlled variation in hand morphology and skin tone, enabling models to better adapt to demographic and anatomical differences. Data accessibility is achieved through simulation-based generation, which circumvents the high costs, ethical concerns, and privacy limitations associated with collecting clinical data. Together, these attributes enable the reproducible creation of large, diverse, and ethically neutral datasets, forming a sustainable foundation for training and benchmarking acupoint localization models. Moreover, the results demonstrate that anatomically precise synthetic data can effectively bridge performance gaps often observed in small or demographically biased real-world datasets.

The controlled experimental design of this study was essential for fair benchmarking, but it introduced several limitations. The dataset focused on five hand acupoints, mirroring the PK dataset to ensure one-to-one comparison. Future work will expand coverage to include forearm, foot, and whole-body acupoints, enabling broader clinical applicability. The dataset also captured only quasi-static postures under fixed lighting and camera geometry; while this ensured comparability, it limits the ecological validity. Subsequent versions of MAP will integrate scene-level domain randomization, including variable lighting, shadows, occlusion, and multi-angle capture to simulate realistic clinical imaging conditions.

The generalization analysis (Experiment 4) evaluated robustness across camera heights but did not incorporate dynamic gestures or motion-based occlusions. Future extensions will therefore include temporal and motion-based data, enabling real-time model evaluation and video-based tracking of acupoints. Moreover, while HRNet-W48 was chosen to isolate dataset effects, ongoing work will examine cross-model transferability using lightweight and transformer-based architectures (e.g., RTMPose, TokenPose) to generalize beyond the current backbone.

To further close the gap between synthetic and real domains, future research will include domain adaptation and style transfer methods (e.g., adversarial fine-tuning or appearance-based adaptation). These techniques will enable the model to better align with both synthetic and clinical data, thereby supporting its application in various clinical and educational settings.

Beyond computer vision benchmarking, MAP serves as a critical link between synthetic data generation and immersive Virtual Reality (VR) applications. By embedding all acupoint coordinates within an anatomically accurate 3D skeletal model, the dataset seamlessly integrates with VR-based clinical training and therapeutic simulators. These anatomically grounded annotations facilitate interactive acupoint visualization, real-time feedback, and gesture-driven learning for students and practitioners of TKM. Moreover, the Unreal Engine–based procedural generation pipeline dynamically renders hand movements and acupoint activations, fostering the creation of immersive, data-driven VR systems. Future developments will emphasize integrating MAP into VR frameworks to enable AI-supported, experiential clinical education.

In conclusion, this study offers strong empirical evidence that high-fidelity synthetic datasets, when crafted with anatomical realism and controlled diversity, can effectively replace or supplement real-world clinical data. MAP demonstrates benchmark-level accuracy comparable to real datasets, improved generalization across unseen imaging conditions, notable enhancements in annotation consistency, and scalability for diverse and ethically responsible data generation. Overall, these results help integrate synthetic data into biomedical AI, creating a reproducible and expandable foundation for acupoint localization and related clinical imaging tasks. Additionally, the modular design of the MAP pipeline supports future growth toward dynamic motion capture, multi-view realism, and immersive VR-based training environments, opening the door for accessible, versatile, and clinically relevant synthetic intelligence frameworks in healthcare.

## Figures and Tables

**Table 1:** Comparative summary of existing datasets and prior studies on acupoint localization and synthetic data generation.

Study/Dataset	Model/Method Used	Real Dataset	Synthetic Dataset	Annotation Consistency	Clinical Evaluation	Domain Randomization
Seo et al. [5]	HRNet-W48 vs. ResNet	✓	✗	Expert-based	✓	✗
Yang et al. [20] (RT-DEMT)	Mamba + Transformer (RT-DEMT)	✓	✗	Not specified	✓	✗
Yang et al. [19]	Self-attention CNN	✓	✗	Not specified	✓	✗
Sun et al. [10]	YOLOv5 + HRNet Cascade	✓	✗	Not specified	✓	✗
Hasan et al. [15] (Hi5)	CNN on synthetic joint annotations	✗	✓	Automated	✗ (Pose only)	✓
Sun et al. [4] (AcuSim)	CNN on RGB-D synthetic data	✗	✓	Automated	✓	Partial
Benmahdjoub et al. [24]	Marker detection using synthetic avatars	✗	✓	Not specified	✗	✓
Tremblay et al. [32]	Domain randomization strategy	✗	✓	✗	✗	✓
Schülein et al. [31]	Simulation + real fine-tuning	✓	✓ ^α^	✗	✓	✓
Khosravi et al. [22]	CNN classifiers	✓	✓ (Augment only)	✗	✗	✗
Kazeminia et al. [8]	GAN-based image synthesis	✓	✓	✗	✗	Partial
MetaAcuPoint (This study)	HRNet-W48 on synthetic and real data	✓	✓	Perfect auto-label	✓ (Quantitative + Qualitative)	✓ (Pose + Demographic)

✓ denotes studies that were included; ✗ denotes studies that were not included. ^α^ Used with real data fine-tuning.

**Table 2:** Reproducibility configuration for synthetic data generation.

Component	Specification
Software & assets	Unreal Engine 5.4.x; MetaHuman Creator presets (no manual albedo edits)
Scene	Table (80 × 45 × 1) cm at world origin; room height 3 m; surface roughness 0.9, specularity 0.1
Lighting	3 × 3 point-light grid, ~1500 lux per light; ambient intensity 0.15
Camera	Top-down at (0, 0, 100 cm); sensor 16 × 9 mm; focal length 35 mm; aperture f/2.5; resolution 2048 × 1152 PNG (fixed)
Animation & capture	Wrist/elbow ±10°; finger flex/extend ±10°; 1 fps for 15 s per hand → 15 frames/hand × 60 hands = 900 images
Annotation	Five acupoint sockets in bone-local coordinates; projected 3D→2D for pixel labels (COCO format)
Down sampling	Output images resized to 1488 × 837 to match PK dataset resolution.
Determinism	Fixed project seed controlling avatar ordering, initial pose offsets, and captured frame indices

**Table 3:** Summary of experimental conditions.

Experiment	Training Set	Testing Set	Goal
Benchmark	PK	PK	Benchmark baseline (4.81 px)
Exp 1	MAP	PK	Validate the synthetic dataset
Exp 2	PK + MAP (760 + 760)	PK	Assess augmentation impact
Exp 3	Auto (MAP) vs. Manual (MAP*) Synthetic Labels	PK	Evaluate annotation consistency
Exp 4	PK and MAP Models	Viewpoint-varied test sets	Assess cross-view generalizability

MAP*: Manually annotated synthetic dataset.

**Table 4:** Point-wise and overall MDEs (pixels) for four training configurations.

PK: MAP	TE3	LI4	TE5	LI10	LI11	Average
100:0 ^α^	3.78 ± 2.03	3.53 ± 1.80	5.68 ± 3.46	6.22 ± 4.09	4.87 ± 2.88	4.81 ± 2.85
0:100	5.01 ± 2.84	4.06 ± 1.98	5.81 ± 3.17	7.44 ± 4.30	6.03 ± 3.35	5.67 ± 3.13
100:100	3.75 ± 2.13	3.60 ± 1.84	5.70 ± 3.64	6.60 ± 4.21	5.10 ± 2.89	4.95 ± 2.94
0:100 ^β^	9.35 ± 6.96	9.68 ± 6.82	12.97 ± 9.53	17.76 ± 12.08	14.03 ± 9.32	12.76 ± 8.94

^α^ Benchmark, ^β^ MAP* dataset.

**Table 5:** Overall MDE (Mean ± SD in mm) for each model on the four camera-height test sets.

Camera Height (cm)	Mean ± SD (mm)		*p*-Value
	PK	MAP	
30	12.82 ± 3.76	6.45 ± 1.69	<0.001
40	10.84 ± 5.04	6.43 ± 1.61	<0.001
50	10.63 ± 4.28	5.16 ± 1.19	<0.001
60	15.80 ± 4.31	5.84 ± 1.86	<0.001

**Table 6:** Statistical summary for camera-height robustness.

Model	F (ANOVA)	*p*-Value	Interpretation
PK	92.6	<0.001	Significant height-dependent variation in error
MAP	3.42	0.017	Minor variation; performance nearly invariant to height

## Data Availability

The data that support the findings of this study are available from the: MetaAcuPoint dataset, DOI: https://doi.org/10.5281/zenodo.17713204.
